# Fine mapping and RNA-Seq unravels candidate genes for a major QTL controlling multiple fiber quality traits at the T_1_ region in upland cotton

**DOI:** 10.1186/s12864-016-2605-6

**Published:** 2016-04-19

**Authors:** Dexin Liu, Jian Zhang, Xueying Liu, Wenwen Wang, Dajun Liu, Zhonghua Teng, Xiaomei Fang, Zhaoyun Tan, Shiyi Tang, Jinghong Yang, Jianwei Zhong, Zhengsheng Zhang

**Affiliations:** Engineering Research Center of South Upland Agriculture, Ministry of Education, Southwest University, 400716 Chongqing, People’s Republic of China

**Keywords:** Fiber quality, Trichome, Fine mapping, Quantitative trait loci (QTL), RNA-Seq, G*ossypium hirsutum* L.

## Abstract

**Background:**

Improving fiber quality is a major challenge in cotton breeding, since the molecular basis of fiber quality traits is poorly understood. Fine mapping and candidate gene prediction of quantitative trait loci (QTL) controlling cotton fiber quality traits can help to elucidate the molecular basis of fiber quality. In our previous studies, one major QTL controlling multiple fiber quality traits was identified near the T_1_ locus on chromosome 6 in Upland cotton.

**Results:**

To finely map this major QTL, the F_2_ population with 6975 individuals was established from a cross between Yumian 1 and a recombinant inbred line (RIL118) selected from a recombinant inbred line population (T586 × Yumian 1). The QTL was mapped to a 0.28-cM interval between markers HAU2119 and SWU2302. The QTL explained 54.7 % (LOD = 222.3), 40.5 % (LOD = 145.0), 50.0 % (LOD = 194.3) and 30.1 % (LOD = 100.4) of phenotypic variation with additive effects of 2.78, −0.43, 2.92 and 1.90 units for fiber length, micronaire, strength and uniformity, respectively. The QTL region corresponded to a 2.7-Mb interval on chromosome 10 in the *G. raimondii* genome sequence and a 5.3-Mb interval on chromosome A06 in *G. hirsutum*. The fiber of Yumian 1 was much longer than that of RIL118 from 3 DPA to 7 DPA. RNA-Seq of ovules at 0 DPA and fibers at 5 DPA from Yumian 1 and RIL118 showed four genes in the QTL region of the *G. raimondii* genome to be extremely differentially expressed. RT-PCR analysis showed three genes in the QTL region of the *G. hirsutum* genome to behave similarly.

**Conclusions:**

This study mapped a major QTL influencing four fiber quality traits to a 0.28-cM interval and identified three candidate genes by RNA-Seq and RT-PCR analysis. Integration of fine mapping and RNA-Seq is a powerful strategy to uncover candidates for QTL in large genomes.

**Electronic supplementary material:**

The online version of this article (doi:10.1186/s12864-016-2605-6) contains supplementary material, which is available to authorized users.

## Background

Cotton is the world’s leading natural fiber and second most valuable oil crop [1]. The cotton fiber, a seed borne epidermal trichome, is a model system for the study of cell elongation and cell wall and cellulose biosynthesis [2]. On the day of anthesis, cells of the ovular epidermis have already been determined to become trichomes, and subsequently undergo elongation, secondary cell wall synthesis and maturation, which are overlapping steps in a complex developmental process [2]. Although many studies have focused on identification of key genes controlling fiber development at different developmental phases [3–5], the molecular mechanisms of fiber development are still not fully understood.

DNA markers provide a powerful tool to study molecular mechanisms underlying complex traits, and facilitate an effective strategy for crop improvement marker-assisted selection (MAS). Over the last decade, at least 1,075 quantitative trait loci (QTL) from 58 studies of intraspecific *G. hirsutum* and 1,059 QTL from 30 studies of *G. hirsutum* × *G. barbadense* populations have been published, for yield, fiber and seed quality, and biotic and abiotic stress tolerance [6]. However, these QTL are localized to large genomic regions that provide only coarse resolution for MAS in cotton breeding, and may include hundreds or even thousands of genes. To precisely select for target genes with a minimum of ‘linkage drag’ from nearby undesirable alleles requires fine-mapping of QTL, and preferably identification of candidate genes.

Due to the complexity of the tetraploid cotton genome, few studies of QTL fine mapping have been reported [7–9]. Cotton genome sequencing [10–14] has provided a rich source of DNA markers for fine mapping, and made it routine to predict QTL candidate genes.

Besides DNA marker technology, technological developments in high-throughput sequencing also offer new opportunities to elucidate mechanisms underlying complex traits. Considerable research has been conducted on the global molecular and biochemical processes underlying fiber development through expressed sequence tag (EST) analysis [3, 15], macro- or microarray gene expression profiling [16, 17] and transcriptome analysis [18, 19]. These studies have highlighted the stage-specific transcription of genes involved in fiber initiation, elongation and secondary cell wall formation. For example, *GhMYB25* [20], *GhMYB25-like* [21], *GhSusA1* [22], *GbPDF1* [23], *GhHD-1* [5], *GhFLA1* [24] and *GhVIN1* [25] have been demonstrated to have definite roles in cotton fiber initiation, and *Sus* [26], *ACTIN1* [27], *GhSusA1* [22], *GhPIP2* [28], *WLIM1a* [29], *GhFLA1* [24], *GhHOX3* [30], *GhCaM7* [31] and *GhPAG1* [32] play roles in controlling cotton fiber elongation.

Rich information about cotton QTL and expanded scope for fine mapping, together with a growing body of developmental and transcriptomic information, sets the stage for unraveling relationships between specific genes and empirically-measured fiber quality traits such as fiber length, strength, fineness, and elongation. Many studies showed that the integration of quantitative genetics and transcriptomic data was very helpful to propose short lists of candidate genes in plants, for example in *Populus spp*, *Glycine max* L. and *Triticum aestivum* L. [33–35].

In our previous studies, one QTL affecting cotton lint percentage, fiber length, uniformity, strength and micronaire was identified near the T_1_ locus on chromosome 6 affecting leaf pubescence [36]. The QTL was initially identified using an F_2_ population (T586 × Yumian 1) in upland cotton and confirmed in a recombinant inbred line population (T586 × Yumian 1) in multiple environments [36, 37]. The T_1_ allele was associated with short and coarse fibers, increased micronaire and high trichome density on the vegetative parts of plants [38–41]. A recent study linked the absence of stem trichomes of *G. barbadense* to a *copia-like* retrotransposon insertion in a homeodomain leucine zipper gene (*HD-1*), which was found to co-segregate with T_1_ on chromosome 6 [42]. Silencing of *GhHD-1* reduced trichome formation and delayed the timing of fiber initiation. Over expression of *GhHD-1* increased the number of fibers initiating on the seed and thereby increased fuzz percentage, but did not affect fiber quality traits [5]. These results suggested that the gene/s for the QTL near the T_1_ locus and the gene for T_1_ itself might not be the same.

In the present study, a large segregating population was established from a cross between Yumian 1 and a recombinant inbred line (RIL118) with trichomes and short coarse fiber selected from a recombinant inbred line population (T586 × Yumian 1). SSR markers were designed from the *G. raimondii* genome [10] for fine mapping the QTL controlling multiple fiber quality traits in the T_1_ locus region. Digital gene expression profiling was used to identify candidate genes for the QTL controlling fiber quality traits.

## Methods

### Mapping population development and fiber quality measurement

Based on its genotype and the location of the QTL mapped in the recombinant inbred line population (T586 × Yumian 1) [37, 43], one recombinant line, RIL118, with trichomes and short and coarse fiber, was selected to cross with Yumian 1 in the summer of 2010 at the Teaching and Experiment Farm of Southwest University (SWU), Beibei, Chong qing, China. Chromosome 6 of RIL118 was homozygous for T586 alleles, and the other chromosomes with loci affecting fiber quality (like N_1_, Lc_1_ and Lg) are homozygous for Yumian 1 alleles. F_1_ plants were self-pollinated in the winter of 2010 in Sanya, Hainan, China. A total of 6975 F_2_ individuals, including 1434 plants in 2011 and 5541 plants in 2012, were planted at SWU. For the 1434 individuals planted in 2011, all naturally-opened bolls were hand-harvested to gin fiber for fiber quality measurement, including fiber length (FL, mm), fiber micronaire reading (FM), fiber strength (FS, cN/tex), fiber elongation (FE), and uniformity ratio (FU). For the 5541 individuals planted in 2012, only the recombinants were sampled for fiber quality measurement. Fiber samples were measured using HVI (High Volume Instrument) at the Supervision Inspection and Testing Cotton Quality Center, Anyang, China. The correlation coefficient among fiber quality traits was determined using Statistical Analysis Software (SAS, Cary, NC).

### Trichome phenotypes

**Fig. 1:**
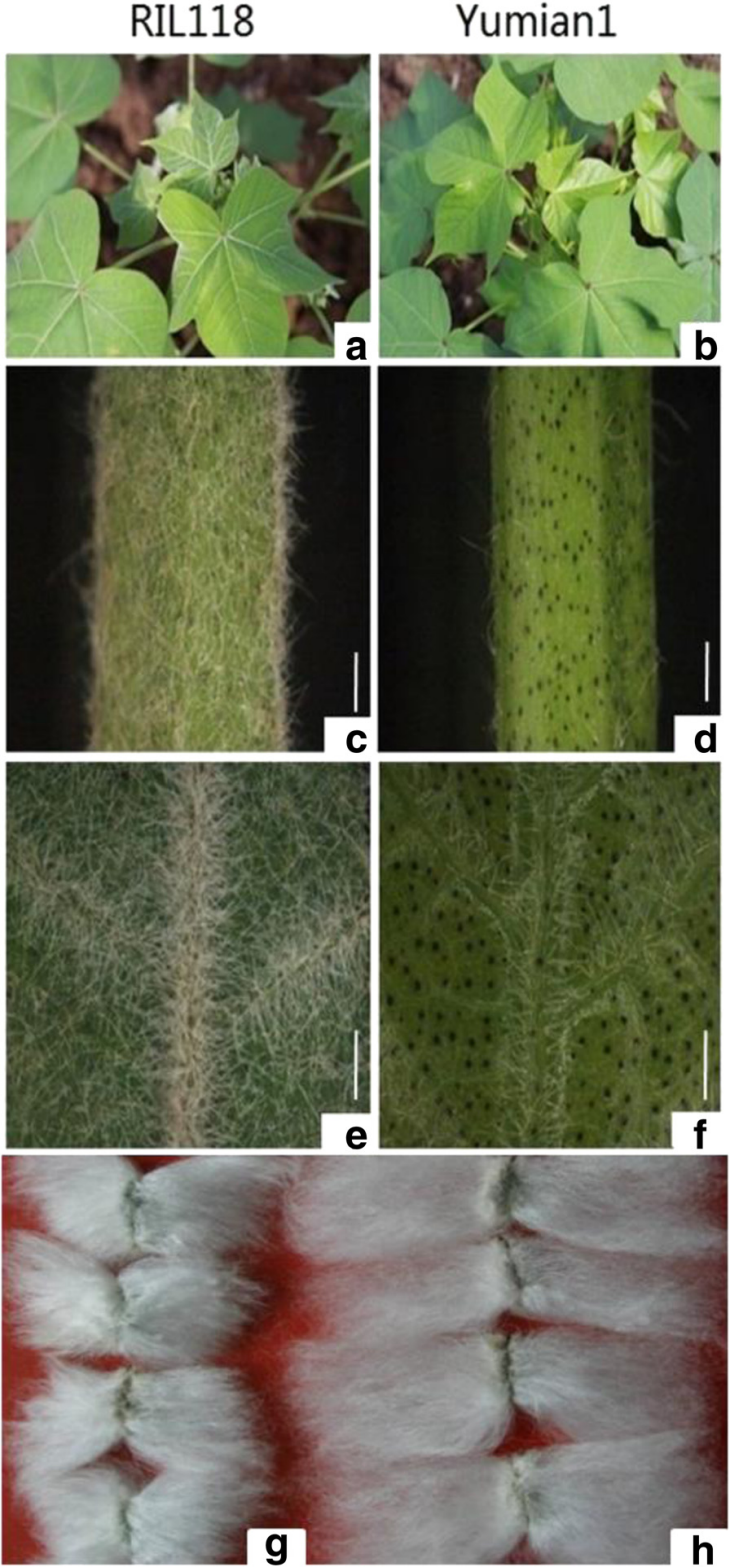
Phenotypeof trichome and fiber traits in *G. hirsutum* RIL118 and Yumian 1. **a**,**c**, **e** and **g** indicate plant, stem, leaf and fiber of RIL118; **b**, **d**, **f** and **h** indicate plant, stem, leaf and fiber of Yumian 1. Scale bars:1 mm (**b**-**e**)

Trichome phenotypes were determined qualitatively by viewing young leaves and stems. The trichome phenotypes for RIL118 and Yumian 1 are shown on Fig. 1. Plants from segregating populations grown in the field were classified into three categories i.e., pilose (TT), semi-hairy (Tt) or glabrous (tt), on the basis of mean trichome density, compared with homozygotes for the parental types.

### Fiber development observation

Yumian 1 and RIL118 were grown in 2014 at SWU. Flowers were tied up the day before anthesis to ensure self-pollination. Young bolls were harvested at 0, 1, 3, 5, and 7 DPA. For scanning electron microscopy, samples (0 and 1 DPA) were prepared as described [44]. The developing cotton ovules were examined and photographed with a Hitachi S-3000 N scanning electron microscope. To monitor the process of fiber elongation for the two parents, an anatomy microscope (Leica, Germany) was used to observe fiber length at 3, 5 and 7 DPA in 95 °C water for 5 min.

### Genetic map construction

Cotton genomic DNA was extracted from young leaves using a modified CTAB method [45].

To enrich markers within the QTL region, three hundred SSR markers were developed from a *G. raimondii* genome sequence [10]. Primers were synthesized by Shanghai Invitrogen, mapped on chromosome 6 [43], and those with clear polymorphism between Yumian 1 and RIL118 were used to genotype the mapping population. JoinMap4.0 was used to construct the genetic map of the T_1_ region. The interval mapping method of MapQTL6.0 was used to identify QTL for the five fiber quality traits. A threshold of log of odds ratio (LOD) ≥3.0 was used to declare QTL. MapChart 2.2 was used to create the linkage group and QTL. QTL were named starting with ‘q’, followed by a trait abbreviation (e.g., FL for fiber length).

### Total RNA isolation

Total RNA was extracted from ovules at 0 DPA and fibers at 5 DPA. RNA degradation and contamination was monitored on 1 % agarose gels. RNA purity was checked with the Nano Photometer spectrophotometer (IMPLEN, Westlake Village, CA, USA). RNA concentration was measured with the Qubit RNA Assay Kit in a Qubit 2.0 Fluorometer (Life Technologies, Carlsbad, CA, USA). RNA integrity was assessed with the RNA Nano 6000 Assay Kit of the Bioanalyzer 2100 system (Agilent Technologies, Santa Clara, CA, USA).

### Library construction and sequencing

At least 3 μg of total RNA per sample was used for RNA sample preparation. Sequencing libraries were generated using Illumina TruSeq™ RNA Sample Preparation Kits (Illumina, San Diego, CA, USA) in accordance with the manufacturer’s recommendations. Transcriptome sequencing was carried out on an Illumina HiSeq 2000 platform that produced 100 bp paired-end (PE) raw reads (Novogene Bioinformatics Technology Co.Ltd).

The raw sequence data (from Illumina HiSeqTM2000/MiSeq) which consisted of raw pictures were first transformed to Sequenced Reads which contained read sequences and corresponding base quality (in FASTQ format) through Base Calling. Raw data (raw reads) was filtered as follows:(1) remove reads with adapter, (2) remove reads containing N > 10 %, (3) remove reads with sQ ≤ 5 base percentage > 50 %. Q20, Q30 and GC content were calculated. All downstream analyses were based on clean data with high quality.

### Mapping clean reads to the reference genome

The clean sequence tags were mapped to the *G. raimondii* reference genome [10]. Gene model annotation files came from Phytozome (https://phytozome.jgi.doe.gov/pz/portal.html). An index of the reference genome was built using Bowtie v2.0.6 and PE clean reads were aligned to the reference genome using TopHat v2.0.9.

### Quantification and pathway in differential expression analysis of transcripts

Gene expression levels were measured by transcript abundance. In our RNA-seq analysis, the gene expression level was estimated by counting the reads that mapped to genes or exons. To make gene expression data comparable across different genes and experiments, the parameter FPKM (Fragments Per Kilo base of exon per Million fragments mapped) was used. HTSeq software was used to analyze gene expression levels, using the ‘union’ model. The result files present the number of genes with different expression levels and the expression levels of single genes.

Because there were no biological replicates, for each sequenced library the read counts were adjusted by the Edger package through one scaling normalized factor. Differential expression analysis of two conditions was performed using the DEGSeq R packagev 1.12.0. DEGSeq provides statistical routines to determine differential expression in digital gene expression data using a model based on the negative binomial distribution. *P*-values were adjusted using the Benjamini and Hochberg method. The q-values of 0.05 and log2 (Fold_change) with no limitations were served as the threshold of significance for differential expression.

Kyoto Encyclopedia of Genes and Genomes (KEGG) is a database resource used to facilitate understanding of the high-level functions and uses of the biological system (http://www.genome.jp/kegg/). Here, KOBAS software was used to test the statistical enrichment of differential expression genes in KEGG pathways.

### Validation of candidate genes by real-time quantitative RT-PCR

Total RNA was extracted from ovules at 0 DPA and fibers at 5, 10,15,20 and 25 DPA of Yumian 1 and RIL118. RNA degradation and contamination was monitored on 1 % agarose gels. First strand cDNA was synthesized from total RNA by priming with oligodT primer using Thermoscript Reverse Transcriptase (Invitrogen, Carlsbad, CA) at 50 °C. RT-PCR was carried out inareaction volume of 20 ml containing 10 ml iTaq^TM^ SYBR®Green Super mix with ROX (Bio-Rad Laboratories), 1 mM forward and reverse primers, and 0.1 mM cDNA template in a quantitative real-time PCR kit (Bio-Rad). PCR reactions were performed according to the manufacturer’s instructions. Cotton HISTONE3 (AF024716) was used as a loading control to normalize samples. Additional file 1 lists the primer sequences of the four candidate genes based on the *Gossypium hirsutum* L*.* reference genome [14].

## Results

### Phenotypic analysis of fiber quality traits

Phenotypic variation for five fiber quality traits was summarized in Additional files 2 and 3. The two parents differed remarkably in these traits, and their F_2_ population of 1434 individuals displayed continuous variation. The average values of fiber quality traits for groups of progeny with different trichome phenotypes are shown in Additional file 4. The fiber quality traits of the T_1_T_1_ genotype were almost exactly the same as RIL118, and those of the t_1_t_1_ genotype were similar to Yumian 1. Additional file 5 showed correlation coefficients for the five fiber traits among 1434 F_2_ plants. All traits had significant correlation with each other. FM had significant negative correlation with FU, FL and FS, whereas significant positive correlations existed among the other traits.

### QTL mapping

**Fig. 2:**
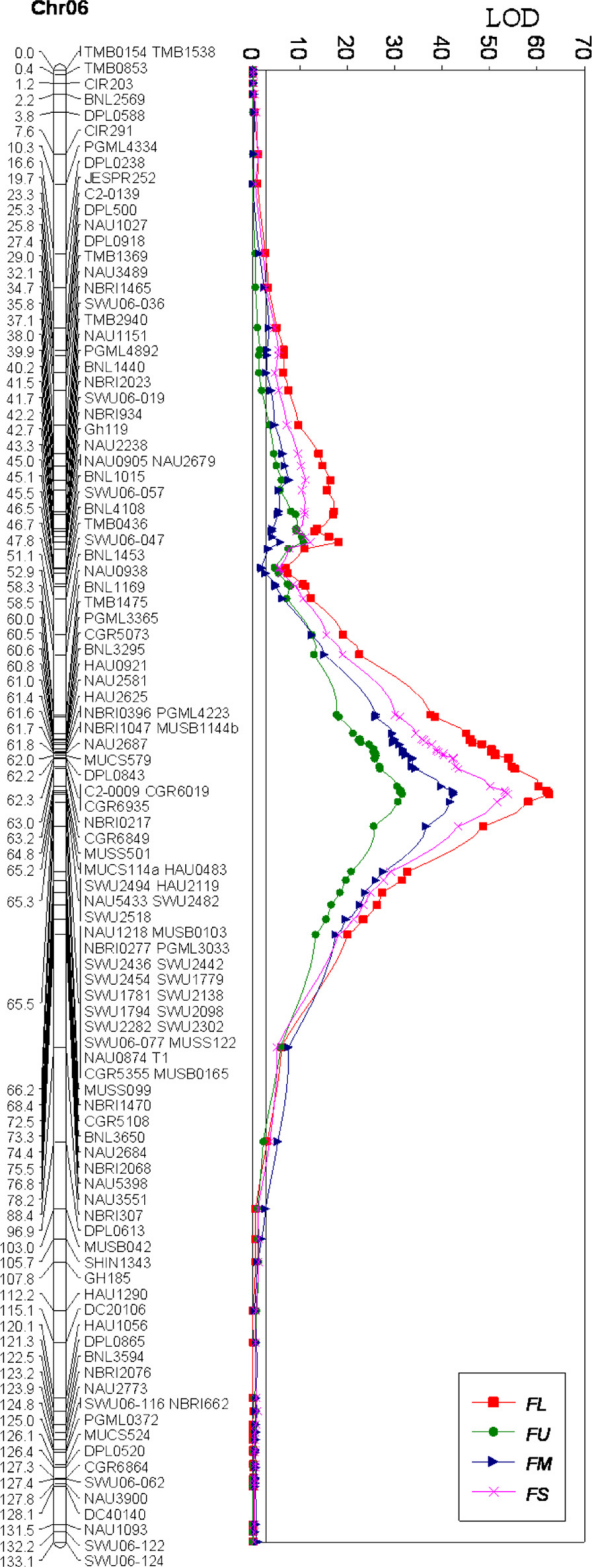
Genetic map and QTL peak map of cotton chromosome 06 from 360 F_2_ plants in 2011

Based on the genetic map and the QTL region for fiber quality traits [37, 43], thirteen newly developed SSR markers from *G. raimondii* reference genome [10] showed polymorphism between Yumian 1 and RIL118 (Additional file 6). The newly identified SSR markers and SSR markers previously mapped on chromosome 6 were used to genotype 360 F_2_ individuals randomly selected from the 2011 F_2_ population, with a total of 116 loci (115 SSR and T_1_) mapped on chromosome 6. The genetic map covered 133.1 cM (Fig. 2). The QTL controlling four fiber quality traits was located in the confidence interval between MUCS114 and MUSS099, and 19 markers co-segregated with T_1_ (Fig. 2 and Table. 1). The QTL explained 59.3 % (LOD = 62.6), 45.7 % (LOD = 42.6), 36.4 % (LOD = 31.6) and 53.8 % (LOD = 52.43) of phenotypic variation, with additive effects of 2.78, −0.43, 1.90 and 2.92 for FL, FM, FU and FS, respectively.

**Table 1:** Biometrical parameters for QTL controlling fiber quality based on coarse mapping

Trait	LOD	A	D	PV (%)
qFL	62.6	2.78	−0.10	59.3
qFM	42.6	−0.43	0.27	45.7
qFU	31.6	1.90	0.28	36.4
qFS	52.4	2.92	−0.33	53.8

*A* is additive effect of the Yumian 1 allele, *D* is dominant effect of the Yumian 1 allele, *PV* is percentage of total phenotypic variance explained by the QTL

### High-resolution mapping

**Fig. 3:**
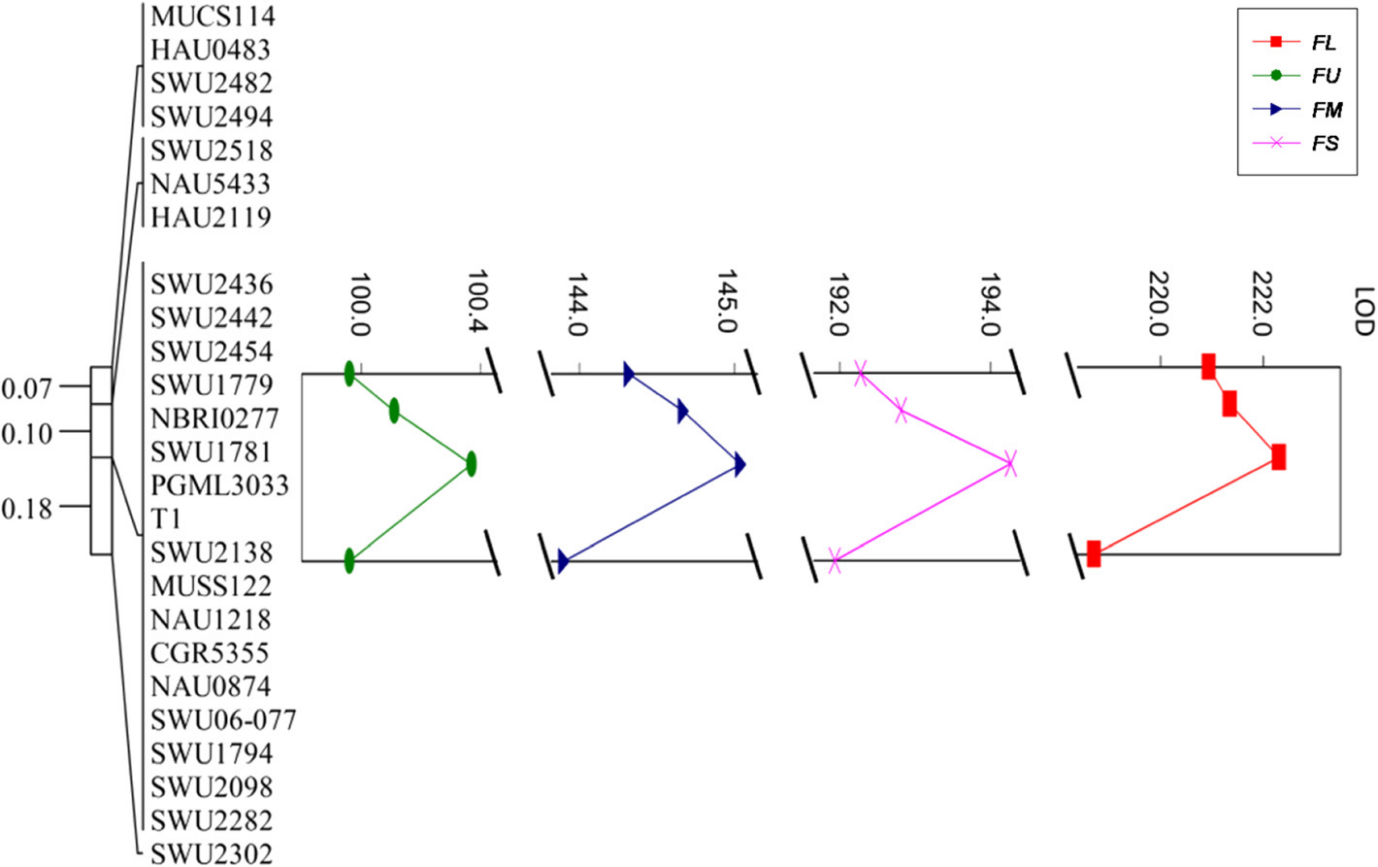
Genetic map of the QTL region and trace of the log probabilities for the four fiber quality traits. The genetic map and QTL identification come from 1440 F_2_ plants in 2011. The genetic distances between adjacent markers are shown to the left of chromosomes

To further define the QTL controlling fiber quality traits, all 26 markers in the confidence interval between MUCS114 and MUSB0165 were employed to genotype the 1434 F_2_ plants in 2011. The confidence interval for the position of the QTL from the peak LOD to less than 1, the QTL controlling fiber quality traits was mapped within a 0.35-cM interval between MUCS114 and SWU2302, and co-segregated with T_1_ and 17 SSR markers (Fig. 3 and Table 2). The QTL explained 54.7 % (LOD = 222.3), 40.5 % (LOD = 145.0), 30.1 % (LOD = 100.4), and 50.0 % (LOD = 194.3) of total phenotypic variation, with additive effects of 2.65, −0.41, 1.83 and 2.91 for FL, FM, FU and FS, respectively.

**Table 2:** Biometrical parameters for QTL controlling fiber quality based on fine mapping

Trait	LOD	A	PV (%)
qFL	222.3	2.65	54.7
qFM	145.0	−0.41	40.5
qFU	100.4	1.83	30.1
qFS	194.3	2.91	50.0

*A* is additive effect of the Yumian 1 allele, *PV* is percentage of total phenotypic variance explained by the QTL

**Fig. 4:**
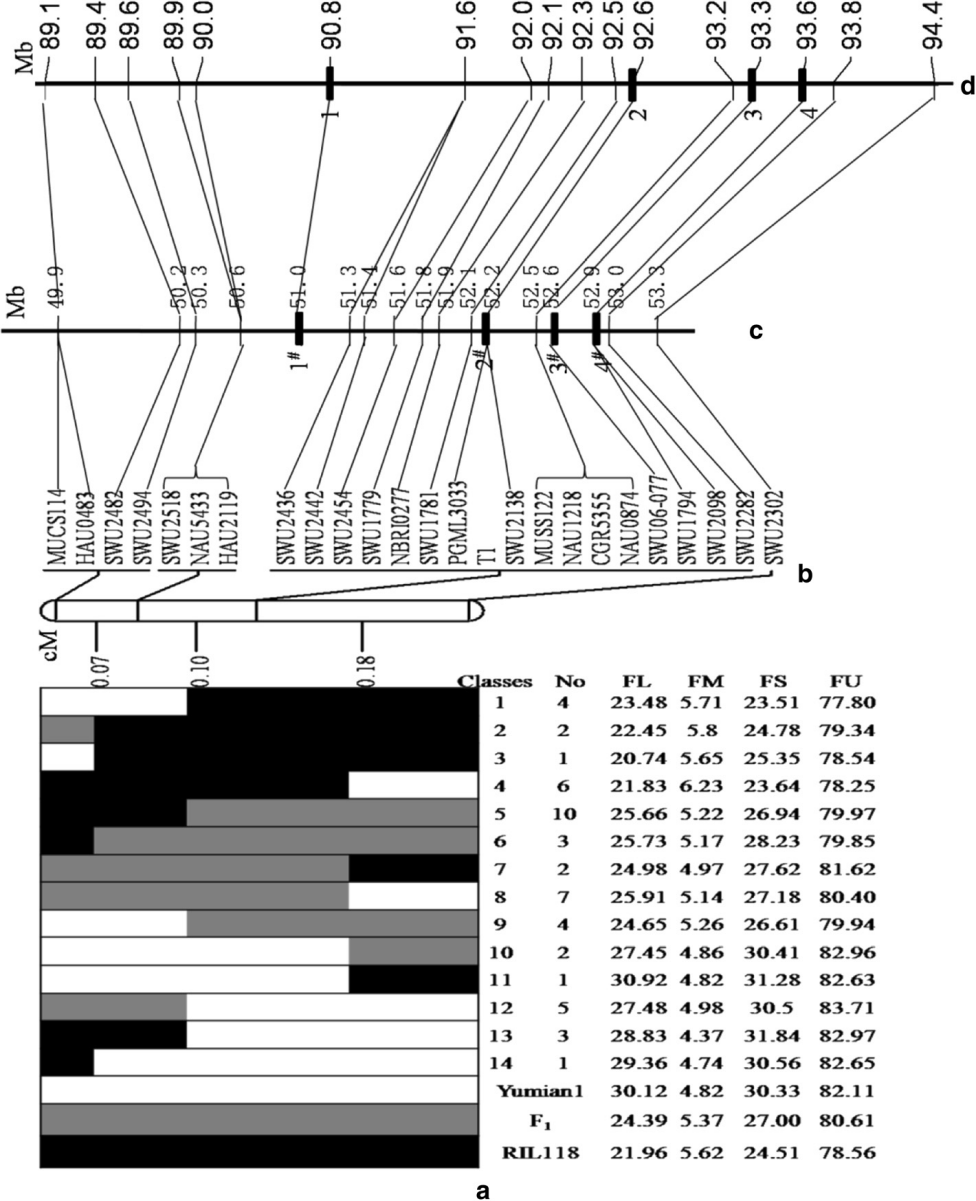
**a** Graphical genotypes and fiber quality traits for recombinants derived from 6975 individuals. White, black and gray bars represent genotypes of Yumian 1, RIL118and heterozygotes, respectively. **b** The genetic map (cM) for the QTL region. **c** The physical locations (Mb) of markers and genes on chromosome 10 of the *G. raimondii* genome sequence and on **d** chromosome A06 of the *G. hirsutum* TM-1 genome sequence. The physical distances of the chromosomes on the plots are represented by cells of different sizes according to the ratio of the chromosome lengths. The candidate genes suggested by RNA-Seq are highlighted with black squares. The figures in turn indicate genes from the *G. hirsutum* genome: *GhA06G1256*, *GhA06G1277*, *GhA06G1301* and *GhA06G1313*. The figures with # in turn indicate genes from the *G. raimondii* genome: *Gorai.010G174800*, *Gorai.010G177300*, *Gorai.010G180100* and *Gorai.010G181500*. The same number of genes indicates the analogous gene of the reference genome

To assess and facilitate genetic mapping, all SSR markers on the genetic map were used to do Blastn searches against *G. raimondii* and *G. hirsutum* genome sequences [10, 14]. All markers could be aligned to the reference genomes, as shown in Fig. 4c, 4d and Additional file 7. The 0.28-cM genetic interval corresponded to a 2.7-Mb physical distance on chromosome 10 in the *G. raimondii* genome and a 4.4-Mb physical distance on chromosome A06 in the *G. hirsutum* genome. Compared to the genome-wide averages of 0.33 Mb per cM for *G. raimondii* and 0.6 Mb per cM for *G. hirsutum* [10, 46], this result suggested that recombination suppression occurred in the region where the QTL located.

### QTL substitution mapping

For further dissection of the QTL controlling fiber quality traits, another 5535 plants were planted in 2012. The two SSR markers MUCS114 and SWU2302 were chosen to screen the recombinants in the QTL region from the 5535 plants. However, no recombination event was detected between T_1_ and the 16 SSR markers, including SWU2436, SWU2442, SWU2454, SWU1779, NBRI0277, SWU1781, PGML3033 SWU2138, MUSS122, NAU1218, CGR5355, NAU0874, SWU06-77, SWU1794, SWU2098 and SWU2282 (Fig. 3). A total of 51 recombinants representing different recombination events between MUCS114 and SWU2302 in the 6975 plants were grouped into 14 classes, and the genotypes of recombinant classes are shown in Fig. 4d. The recombinant groups in the first category included recombinant classes 2, 3, 6 and 14, which placed the QTL downstream of SWU2494. The recombinant groups in the second category included recombinant classes 1, 5, 9, 12 and 13,which placed the QTL downstream of HAU2119. The recombinant groups in the third category included recombinant classes 4, 7, 8,10 and 11, which placed the QTL up stream of SWU2302. Therefore, the QTL controlling fiber quality traits was mapped into a 0.28-cM interval between HAU2119 and SWU2302.

### Comparison of fiber development

**Fig. 5:**
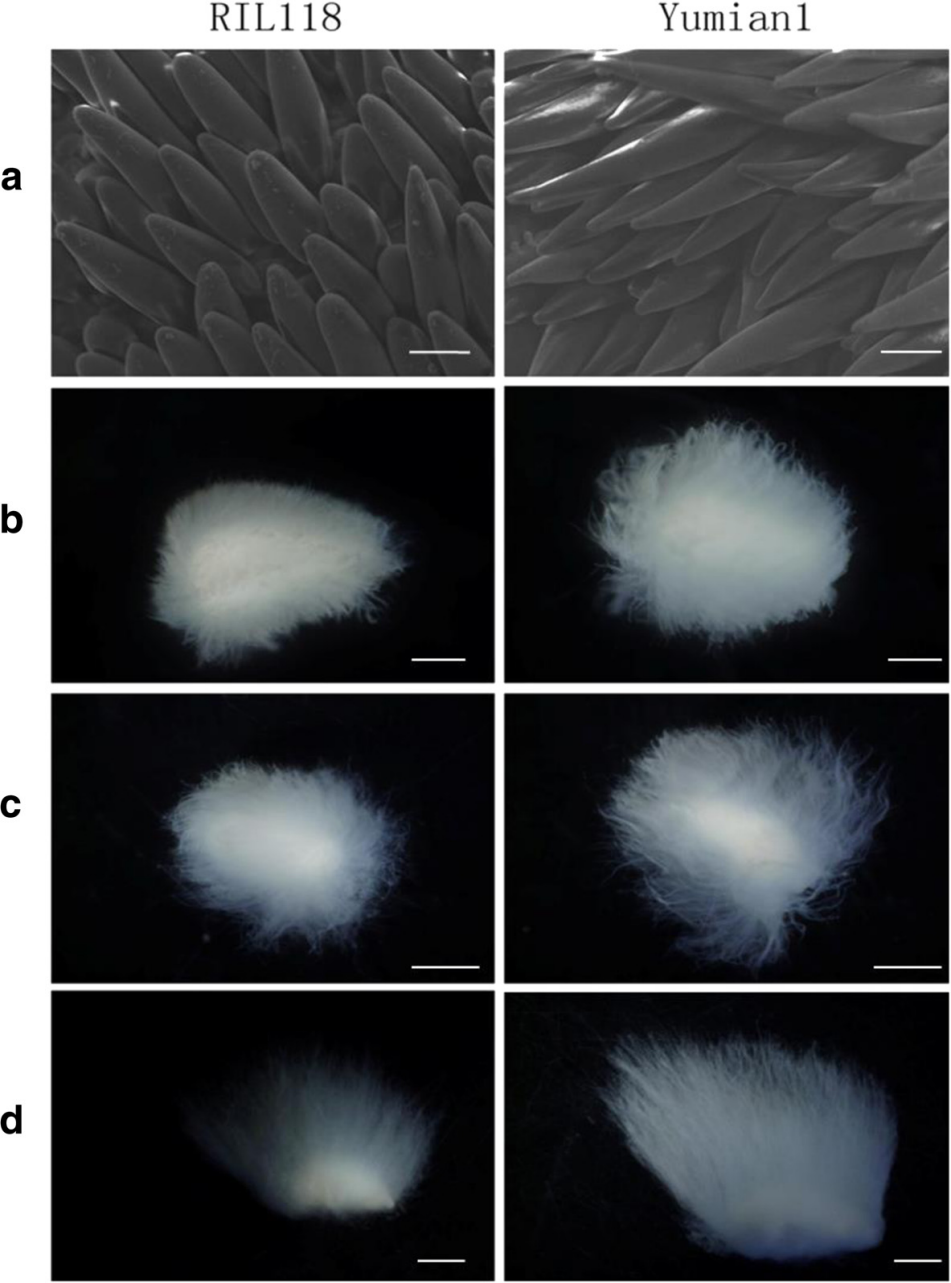
Phenotype valuation during early fiber development. **a**: Scanning electron microscope mages of the +1 DPA ovule; **b**, **c** and **d**: Anatomy microscope images of the +3, +5, +7 DPA fiber. Scanning electron microscope images were taken at a similar position in the middle of ovules. Scale bars: 200 μm (**a**); Scale bars: 1 mm (**b**); Scale bars: 2.5 mm (**c**, **d**)

To examine fiber cell differentiation in Yumian 1 and RIL118, scanning electron microscopy was used to observe the development of fiber cell initials in the ovular surface at 1 DPA, and anatomy microscopy was used to observe progress in fiber cell elongation at 3 DPA, 5 DPA and 7 DPA. The fiber length of Yumian 1 was much longer than that of RIL118 from 3 to 7 DPA (Fig. 5). This result showed that fiber development at an early stage has a positive effect on final fiber quality.

### Identification of QTL candidate genes

To better understand the molecular basis of early fiber development, RNA extracted from 0 DPA ovules and 5 DPA fibers was sequenced using an Illumina Hiseq 2500 platform. An overview of the sequencing and assembly was outlined in Table 3. With the removal of low quality tags, a total of 10 million and 14 million high-quality clean reads were obtained from 0 DPA ovules and 5 DPA fibers mRNA libraries, respectively. Ninety-four percent of the clean reads had Phred-like quality scores at the Q30 level (an error probability of 0.01). Approximately 80 %–83 % of the distinct tags (83–87 % of the total tags) could be mapped uniquely to the *G. raimondii* reference sequence [10], while small proportions (3.5–3.9 %) were mapped to multiple loci in the reference genome (Additional file 8).

**Table 3:** Summary of sequence assembly after Illumina sequencing

Sample name	Raw reads	Clean reads	Clean bases	Error rate (%)	Q20 (%)	Q30 (%)	GC content (%)
R118_0	10144830	9985483	0.5G	0.01	97.94	94.16	43.74
Yumian 1_0	11213370	11081107	0.55G	0.01	98.01	94.35	43.77
R118_5	14141978	14104947	0.71G	0.01	99.03	96.58	44.01
Yumian 1_5	14027353	13969205	0.7G	0.01	98.99	96.49	43.94

Raw reads: Statistical data for the original sequences. Clean reads: Calculation method is the same as for raw reads, but the statistics file is the filtered data. Clean bases:sequence number*sequence length (transformed into G bases). Q20、Q30:Percentage of the number of bases of Phred score greater than 20, 30 respectively. GC content:Percentage of G and C bases among the total base number

**Fig. 6:**
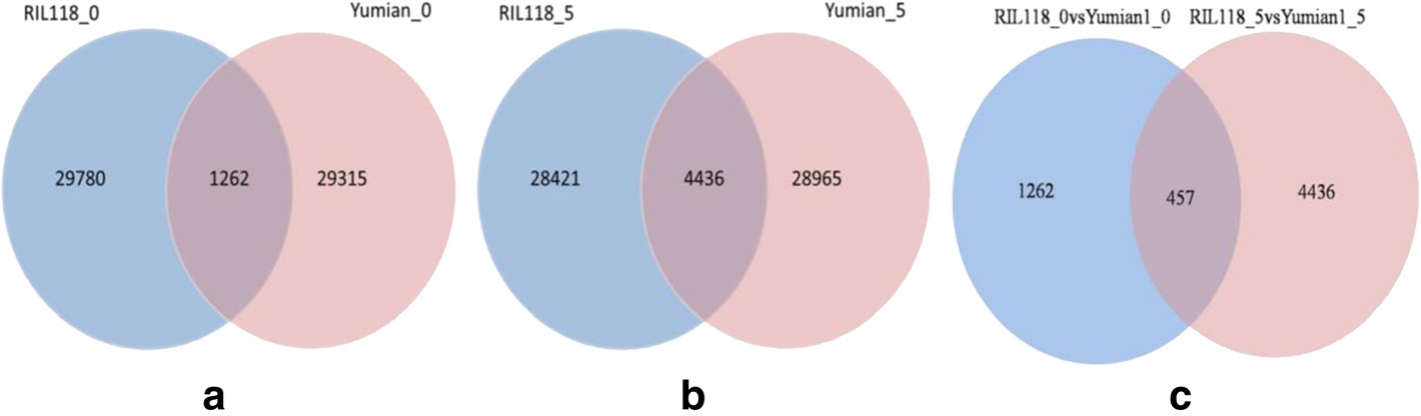
Venn diagram showing the number of differentially expressed genes in ovules and fibers. **a b** Differentially expressed genes between RIL118 and Yuan 1 on 0 DPA ovule and 5DPA fiber; **c** The expression of regulated genes were significantly different between two joint time points (*P*-value < 0.05 and fold change > 1)

The total number of mapped reads for all identified transcripts was used for differential expression analysis in DESeq with |log2 (Fold Change)| > 1 and q value < 0.005. There were 1262 genes with significantly different expression levels between Yumian 1 and RIL118 at 0 DPA ovules, among which 1006 were up-regulated and 256 were down-regulated in Yumian 1 (Fig. 6a). There were 4436 genes with significantly different expression levels between Yumian 1 and RIL118 at 5 DPA fibers, among which 2315 were up-regulated and 2121 were down-regulated in Yumian 1 (Fig. 6b). Combining the two time-points of cotton fiber development, 457 significantly differentially expressed genes were observed with a consistent high-or-low variation between 0 DPA ovules and 5 DPA fibers (Fig. 6c). KEGG pathway-based analysis facilitated systematical study on complicated metabolic pathways and biological behaviors of functional molecules. Among differentially expressed genes comparing RIL118 and Yumian1, there were 107 and 122 pathways determined at the 0 DPA ovule and 5 DPA fiber, respectively. The most 20 pathways significantly enriched genes at two stages were showed in Additional file 9.

Among the transcripts aligned to the *G. raimondii* genome, four extremely differentially expressed genes were found within the QTL region between HAU2119 and SWU2302 on chromosome 10 (corresponding to chromosomes 6 and 25 of tetraploid cotton) (Fig. 4b). These genes are *Gorai.010G174800*, *Gorai.010G177300*, *Gorai.010G180100* and *Gorai.010G181500* in *G. raimondii* (Table 4c), which are putative homologues of *GhA06G1256*, *GhA06G1277*, *GhA06G1301*, *GhA06G1313* in the corresponding *G. hirsutum* region (Fig. 4d and Additional file 7).

**Table 4:** Differentially expressed genes within the interval between SWU2302 and HAU2119

Gene ID	FPKM				log_2_FC_0	log_2_FC_5	Gene annotation
	R118_0	Yumian1_0	R118_5	Yumian1_5			
Gorai.010G174800	298.9	110.3	2083.4	74.0	1.4	4.8	AL7B4
Gorai.010G177300	1.49	19.9	4.1	24.5	−3.8	−2.6	XTH_8
Gorai.010G180100	68.6	152.4	69.0	138.2	−1.2	−1.0	UGPI6
Gorai.010G181500	42.1	106.80	49.6	106.2	−1.3	−1.1	GCST

*FPKM* fragments per kilo base of exon per million fragments mapped, *FC* fold changes

**Fig. 7:**
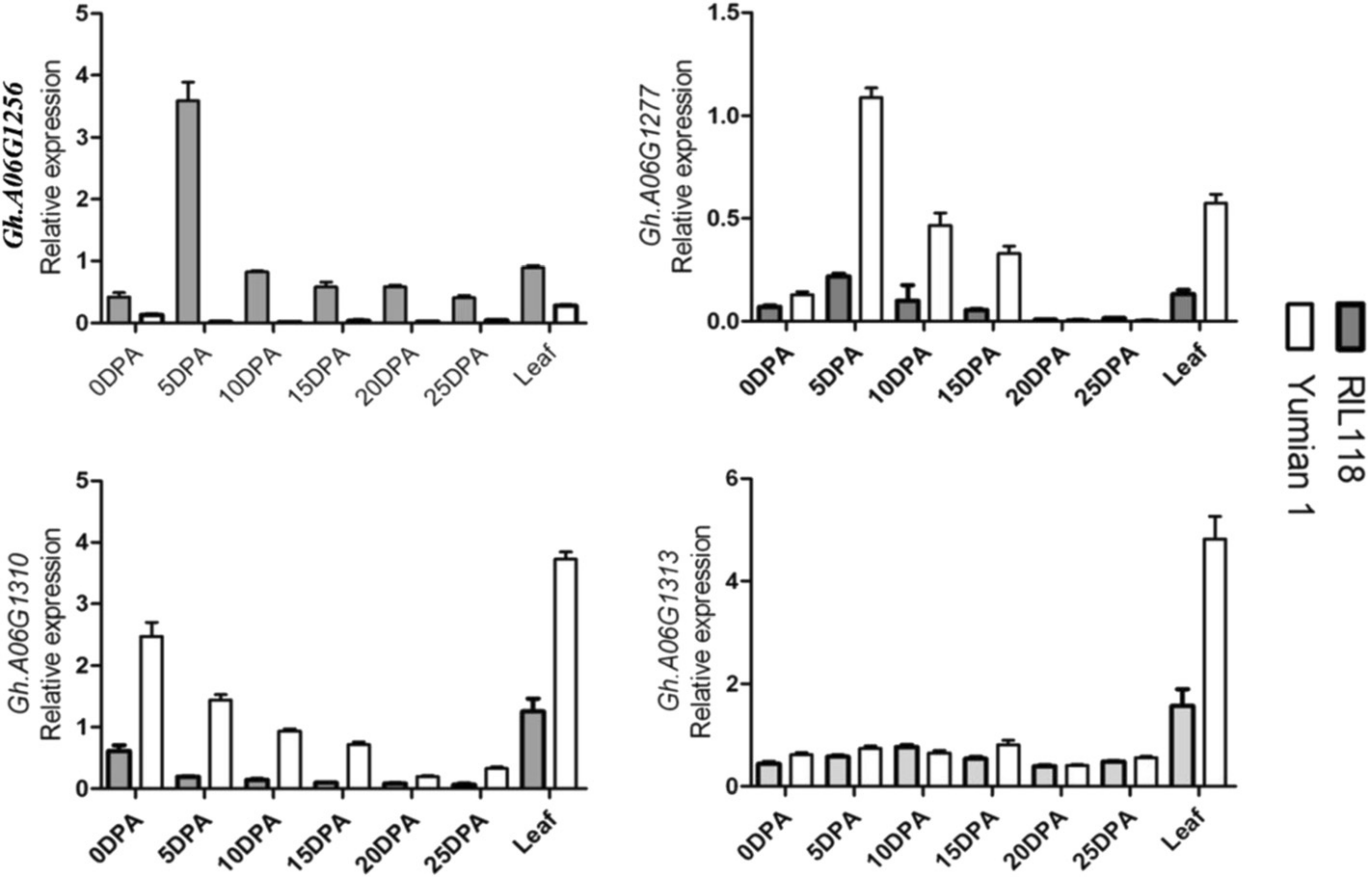
RT-PCR expression of the differentially expressed genes in leaf and during fiber development of RIL118 and Yumian1. All data were normalized to the expression level of actin. Error bars indicate standard deviation of three biological replicates

To confirm whether the digital gene expression results were reliable, and to investigate the activation of four differentially expressed genes in the QTL region, we further tested the four genes using qRT-PCR in young leaf and fiber tissues across six time-points, designing primers from the A-subgenome of *G.hirsutum* [14]. The qRT-PCR analysis demonstrated that three of the four genes were expressed in a manner consistent with the RNA-Seq results (Fig. 7). Among them, *GhA06G1256*, homologous to *Gorai.010G174800*, exhibited a dramatic increase in 5 DPA fiber of RIL118. *GhA06G1277*, homologous to *Gorai.010G177300* was up-regulated in Yumian 1. *GhA06G1301*, homologous to *Gorai.010G180100*, had higher transcript levels in Yumian 1 than inRIL118. However, the qRT-PCR result for *GhA06G1313*, homologous to *GhA06G1313* homologous to *Gorai.010G181500*, was not entirely consistent with RNA-Seq. The RNA-Seq data for this gene at different fiber development points was not supported well by qRT-PCR analysis. RNA-Seq data analysis based on the *G. raimondii* genome may reflect expression of both A and D subgenome-derived loci in *G. hirsutum*, and qRT-PCR data may only reflect the expression of A subgenome genes of *G. hirsutum*.

## Discussion

### Major QTL affecting fiber quality traits at T_1_ locus

T_1_ imparts heavy pubescence on the vegetative parts of cotton and is associated with short, coarse fibers [38]. Several workers [47, 48] have backcrossed t_1_t_1_ into T_1_T_1_ lines, and none have broken the short and coarse fiber in T_1_T_1_ plants without selection. In order to further understand the relationship between T_1_ and fiber traits, Kloth found that the T_1_ marker accounted for 10-75 % of the phenotypic variation associated with seven fiber traits, and suggested that the T_1_ locus is linked to (or pleiotropic with) numerous loci that influence fiber traits.

Based on a RFLP map of QTLs affecting density of leaf and stem trichomes, Wright et al. first mapped the T_1_ locus on chromosome 6, and Lacape et al. and Guo et al. [49] later reported pubescence to map to the same region. QTL for leaf and stem pubescence [50], and QTL for FL, FM, FE, and FU [51–53] have also been mapped to the T_1_ region on chromosome 6 in tetraploid cotton. Our previous studies mapped T_1_ and the QTL for fiber-related traits between marker BNL3650 and BNL4108 on chromosome 6 [36, 37, 45]. The present study mapped T_1_ and the QTL controlling fiber quality traits to a 0.28-cM interval. These results suggested that a major QTL affecting fiber quality traits exists at, or very near, the T_1_ locus.

### Recombination suppression

In the present study, T_1_ and the QTL controlling fiber qualities were mapped into a 0.28-cM interval between HAU2119 and SWU2302 on chromosome 6 from a F_2_ population with 6975 individuals. The 0.28-cM interval corresponded to a 2.7-Mb physical distance on chromosome 10 in the *G. raimondii* genome [10], and a 5.3-Mb physical distance on chromosome A06 in the *G. hirsutum* genome [14]. The average ratio of physical-to-genetic distance is about 600 kb/cM in tetraploid cotton [46]. This result indicated that substantial recombination suppression occurred in the region where the QTL was located. The recombination suppression might come from the origin and position of the T_1_ region. T_1_ region might come from Hawaiian wild tetraploid cotton (*G. tomentosum*) [54, 55], and also might be adjacent to the centromere. The centrome reregion is often associated with depression of meiotic recombination [56]. Recombination suppression was also found on chromosome 24 with a major QTL controlling fiber qualities from the population with line 7235 as one mapping parent, whose chromosome 24 might come from *G. barbadense* or *G. anomalum* [57–59]. The suppressed recombination in these regions suggests that positional cloning of the QTL causal gene may be very challenging.

### Identification of candidate genes for QTL controlling fiber quality traits

Our study showed that fiber length of Yumian1 was much longer than that of RIL118 at early development stages (from 1 to 7 DPA). RNA-Seq and qRT-PCR analysis showed that only three genes *GhA06G1256*, *GhA06G1277* and *GhA06G1301* in the QTL region were differentially expressed in the fiber of Yumian 1 and RIL118. *GhA06G1256* encodes a superfamily of aldehydedehydrogenases (ALDH), which can oxidize many endogenous aromatic and aliphatic aldehydes, creating carboxylic acids [60]. Family 7 aldehydedehydrogenase genes were essential for responsiveness to osmotic stress in leaves and seeds. Fiber development seemed to be concerned with changes in cell turgor pressure [61] and achieved expansion through the influx of water driven by a relatively high concentration of osmoticum within a cell [62]. Our result was consistent with the report that the expression of *GhA06G1256* decreased gradually in the fiber of wild type cotton but is just the opposite in a fuzzless mutant during early development [63]. *GhA06G1277* codes a xyloglucan endotransglycosylase/hydrolase (XTH), an enzyme that catalyzes the cleavage of donor xyloglucan chains and the reconnection of their reducing ends to non-reducing ends of other xyloglucan molecules. Some XTH genes have been reported to loosen cell walls and lead cell expansion and elongation [64]. The relationship of XTH activity and cell elongation has been reported in elongating fiber [65, 66]. It was also observed that XTH activity of wild-type fiber was higher than that of the Li_1_ mutant and the xyloglucan content was lower in wild-type [67]. OsXTH8 was highly expressed in vascular bundles of leaf sheath and young nodal roots where the cells are actively undergoing elongation and differentiation [68]. *GhA06G1301* encodes a plant-specific glycosylphosphatidylinositol (GPI)-anchored protein, and have been found to play roles in primary cell walls and secondary cell walls cellulose biosynthesis. GPI-anchored proteins have various impacts on plant growth including root hair development [69, 70], plant height [71], and pollen development [72]. In further studies, we will clone these candidate genes from the genome and cDNA and investigate the relationship between candidate genes and fiber quality traits through transgenic technology.

### Genetic dissection of complex traits through fine mapping and RNA-Seq

The elucidation of gene and phenotype relationships remains a major challenge in biology. Fine mapping of interesting traits is an important basis for gene or QTL cloning, but experimental approaches are labor-intensive, time-consuming and expensive [73]. In order to reduce the number of candidate genes, here it was possible to form a bridge between the approaches of QTL mapping and transcriptomics [34]. Gilbert et al. [74] and Thyssen et al. [75] mapped candidate genes for the mutants Li_1_ and Li_2_ by the use of genomic and genetic data. In the present study, a QTL controlling fiber quality traits was mapped using a large population (6975 plants), to a 0.28-cM interval corresponding to a 2.7-Mb physical distance on chromosome 10 in the *G. raimondii* genome [10] and a 5.3-Mb physical distance on chromosome A06 in the *G. hirsutum* genome [14]. Although many markers existed in the region, the resolution of QTL mapping could not be improved due to recombination suppression in the region. However, based on expression profiling, four candidate genes were mapped to the QTL region. These studies showed that the combination of fine mapping and RNA-Seq is a powerful strategy to identify candidate genes for QTL controlling complex traits. RNA-seq technology has superior advantages on comparison with gene expression arrays, however, it contains significant blind spots on the gene structure alterations with the same expression level. For the next work, we will clone all the genes and confirm which one is the major QTL controlling fiber quality.

### Fiber quality determined during early fiber development

Through comparing different cotton genotypes or species, insights are emerging about the time in development at which cotton fiber quality is established. Seagull et al. [76] reported that the fibers of *G. barbadense* started out much finer than those of *G. hirsutum* and fiber fineness was mainly determined by their initial diameter. Global gene expression profiling between *G. hirsutum* and *G. barbadense* showed that few meaningful differences were found at the fiber thickening stage, whereas the most significant differences were found at earlier stages of development, which suggested that their different final fiber quality properties may be established at earlier stages of fiber development [77]. Another study showed that targeted expression of the IAA biosynthetic gene *iaaM* in the epidermis of cotton ovules at the fiber initiation stage had increased fiber fineness [78]. Our study showed that fiber length and genes (*ALDH*, *XTH* and GPI-anchored protein) related to fiber elongation were significantly different between long fiber cultivar Yumian 1 and short fiber line RIL118 at the early development stage. These results support the hypothesis that final fiber quality might be determined during early fiber development.

## Conclusion

In summary, a major QTL controlling four fiber quality traits is finely mapped to a 0.28-cM interval at T_1_ region, which correspondes to a 2.7-Mb physical distance on the chromosome 10 in *G. raimondii* genome and a 4.4-Mb physical distance on chromosome A06 in *G. hirsutum* genome. This finding indicates that substantial recombination suppression occurring in the region where the QTL is located. Fiber length of Yumian 1 was much longer than that of RIL118 on 3 DPA and later. RNA-sequencing and RT-PCR analysis showed that three genes are largely expressed between the two parents in the fiber development. These three genes may be candidate genes within the major QTL controlling fiber quality traits.

### Availability of supporting data

The RNA-seq data discussed in this publication have been deposited in the Sequence Read Archive of NCBI under the accession number SRP070870. The data sets supporting the results of this article are included within the article and its additional files.

## Additional files

Additional file 1: Table S1. RT-PCR primer developed from *G. hirsutum* genome sequence. (XLSX 10 kb) Additional file 2: Figure S1. The frequency distribution of five fiber quality traits in 1434-individual F_2_ population in 2011. (DOCX 607 kb) Additional file 3: Table S2. Phenotypic variation of fiber quality traits in 1434 F_2_ population. (XLSX 11 kb) Additional file 4: Table S3. Average value of fiber quality related traits for different trichome phenotype. (XLSX 10 kb) Additional file 5: Table S4. Correlation coefficient among fiber quality traits in F_2_ population. (XLSX 10 kb) Additional file 6: Table S5. New SSR markers developed from *G. raimondii* genome sequence. (XLSX 11 kb) Additional file 7: Table S6. The physical locations of markers and genes on the reference genomes. (XLSX 11 kb) Additional file 8: Table S7. Overview of Mapping Status by RNA-Seq. (XLSX 11 kb) Additional file 9: Figure S2. KEGG analysis results of differentially expressed genes at the 0 DPA ovule and 5 DPA fiber comparing RIL118 and Yumian1. (DOCX 195 kb)

## Abbreviations

DPA: days post-anthesis
FE: fiber elongation
FL: fiber length
FM: fiber micronaire reading
FPKM: fragments per kilo base of exon per million fragments mapped
FS: fiber strength
FU: uniformity ratio
KEGG: Kyoto encyclopedia of genes and genomes
LOD: log of odds ratio
MAS: marker-assisted selection
QTL: quantitative trait loci
RIL: recombinant inbred line
RNA-seq: high-throughput sequencing of RNA
RT-PCR: real-time quantitative PCR
