# Supplementary material for: Fine mapping and RNA-Seq unravels candidate genes for a major QTL controlling multiple fiber quality traits at the T1 region in upland cotton
Source: BMC Genomics. 2016 Apr 19;17:295. doi: 10.1186/s12864-016-2605-6 (PMC4837631; doi:10.1186/s12864-016-2605-6)
Supplement: Additional file 2: Figure S1. — The frequency distribution of five fiber quality traits in 1434-individual F2 population in 2011. (DOCX 607 kb) [file 12864_2016_2605_MOESM2_ESM.docx]

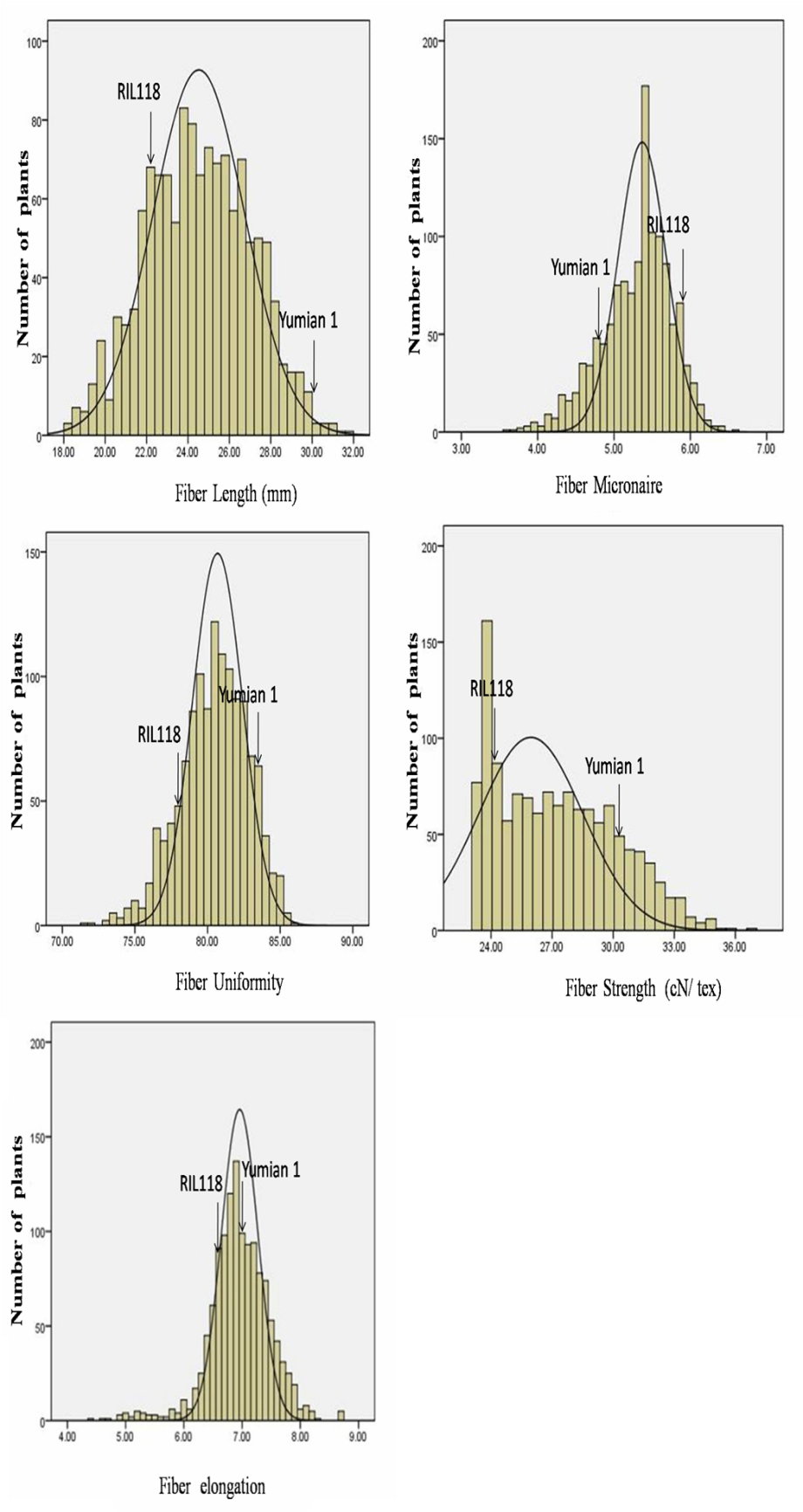


**Figure 1 The frequency distribution of five fiber quality traits in 1434-individual F_2_ population in 2011.**

**Table 1 RT-PCR primer developed from *G. hirsutum* genome sequence**

| Gene ID | Forward 5'>>>3' |
| --- | --- |
| A06G1257F | TGGGCCACAAGGAAGTGACA |
| A06G1257R | AGCCGAAATTGATTCCTTGG |
| A06G1277F | TACCCTGCCTGTGTTTCCAG |
| A06G1277R | CTCCAGGCAATTTAGGATAT |
| A06G1301F | GGTGGTTGGCAAGTAAACTTG |
| A06G1301R | CATTTCCACAGCTATTTGCG |
| A06G1313F | TTGCAGGGAGAATGGTGGAC |
| A06G1313R | AGCCTGCATTCACAACCAGA |

**Table 2 Phenotypic variation of fiber quality traits in 1434 F_2_ population**

| Trait | RIL118 | Yumian 1 | Mean | Max. | Min. | SD | Skewness | Kurtosis |
| --- | --- | --- | --- | --- | --- | --- | --- | --- |
| FL | 21.96 | 30.12 | 24.54 | 31.64 | 18.21 | 2.51 | -0.06 | -0.477 |
| FU | 78.5 | 82.1 | 80.47 | 85.9 | 71.5 | 2.34 | -0.63 | 2.59 |
| FM | 5.62 | 4.82 | 5.29 | 6.62 | 3.55 | 0.52 | -0.41 | 0.09 |
| FE | 6.66 | 7.05 | 6.81 | 8.62 | 4.38 | 2.17 | -0.52 | 2.06 |
| FS | 24.51 | 30.33 | 27.25 | 36.6 | 23.3 | 2.9 | 0.44 | -0.74 |

**Table 3 Average value of fiber quality related traits for different trichome phenotype**

| Genetype | FL | FU | FM | FE | FS |
| --- | --- | --- | --- | --- | --- |
| t_1_t_1_ | 27.28 | 82.11 | 4.82 | 6.68 | 30.35 |
| T_1_T_1_ | 21.97 | 78.44 | 5.63 | 7.00 | 24.51 |
| T_1_t_1_ | 24.39 | 80.61 | 5.37 | 7.08 | 27.00 |

T_1_T_1_: Average value of plants with thick trichome like RIL118 in 1434 F_2_ population in 2011; t_1_t_1_: Average value of plants sparse/normal hairy like Yumian 1 in 1434 F_2_ population in 2011; T_1_t_1_: Average value of plants in middle phenotype in 1434 F_2_ population in 2011.

**Table 4 Correlation coefficient among fiber quality traits in F_2_ population**

| Trait | FL | FU | FS | FM |
| --- | --- | --- | --- | --- |
| FU | 0.55^**^ |  |  |  |
| FS | 0.85^**^ | 0.55^**^ |  |  |
| FM | -0.63^**^ | -0.47^**^ | -0.54^**^ |  |
| FE | -0.28^**^ | -0.12^**^ | -0.41^**^ | 0.18^**^ |

** indicate significance differences with a probability level of 0.01

**Table 5 New SSR markers developed from *G. raimondii* genome sequence**

| Name | Forward 5'>>>3' | Reverse5'>>>3' |
| --- | --- | --- |
| SWU1779 | ATGGGAGTTCGATGCTTGAC | CATGGCCCACTTTGTCTTCT |
| SWU1781 | CTGGAGTTAAGTCCGGAAATG | CCCAAATACCAACAAGATCCA |
| SWU1794 | AGTTGTGGCAGCTTCTTTGG | ATTCCACCACTGCCACTACC |
| SWU2098 | GGCCATGGTAATGGTTATGG | CACCTCCTCCACTAGTCCCA |
| SWU2138 | AGTGGAGTGAACGTATCG | ATTCGTAATGGATACCTTGG |
| SWU2282 | CGATGTCACTGTCTGGTTTGA | CGATACCACATCGAAGATGC |
| SWU2302 | AGAATATTTCATGCATTCCGA | TCCCTTTCAATGTAAGCAAGC |
| SWU2436 | CCTCCTCACGTTTATGATCTCC | AAGGACACACACAGAGATTGTGA |
| SWU2442 | GCTTCTCATGCAAGGGTCTC | TGCGGCATTTGAGATAATAAA |
| SWU2454 | CCTCTTCACCTAATAACCCTTTCA | TGGTGGAGTAAAGGGAAGCC |
| SWU2482 | GCAAACCAAGAAACAGGAGAA | CAATGCTGAGCTTCTTGTCG |
| SWU2494 | CCGTTGCTTACCAGTCCAAG | GCAACTGATGACAACGAGGA |
| SWU2518 | TTGCTGCACCAAATGTCAAT | AGATACTGTTAAAGCGTGAGCC |

**Table 6 The physical locations of markers and genes on the reference genomes**

| Name | Chromosome 10 of *G. raimondii* | Chromosome A06 of *G. hirsutum* |
| --- | --- | --- |
| MUCS114 | 49390321 | 89074367 |
| HAU0483 | 49390448 | 89074511 |
| SWU02482 | 50210275 | 89413327 |
| SWU02494 | 50339475 | 89576889 |
| SWU02518 | 50554503 | 89992438 |
| NAU5433 | 50623024 | 89924397 |
| HAU2119 | 50623241 | 89924834 |
| Gorai.010G174800 | 50947444 | 90765222 |
| SWU02436 | 51340400 | 91606199 |
| SWU02442 | 51397002 | 91641613 |
| SWU02454 | 51618341 | 91992748 |
| SWU01779 | 51828690 | 92074151 |
| Gorai.010G177300 | 51930366 | 92317707 |
| NBRI0277 | 51934207 | 92321371 |
| SWU01781 | 52066519 | 92468142 |
| PGML3033 | 52190850 | 92577038 |
| SWU02138 | 52195863 | 92582047 |
| MUSS122 | 52504990 | 93195954 |
| NAU1218 | 52504997 | 93195961 |
| CGR5355 | 52505138 | 93196143 |
| NAU0874 | 52505142 | 93196106 |
| Gorai.010G180100 | 52608388 | 93284919 |
| SWU06-077 | 52632605 | 93304509 |
| SWU01794 | 52901480 | 93600447 |
| SWU02098 | 52901934 | 93600894 |
| Gorai.010G181500 | 52905674 | 93605874 |
| SWU02282 | 53024776 | 93749032 |
| SWU2302 | 53323195 | 94366568 |

**Table 7 Overview of Mapping Status by RNA-Seq**

| Sample name | R118_0 | Yumian 1_0 | R118_5 | Yumian 1_5 |
| --- | --- | --- | --- | --- |
| Total reads | 9985483 | 11081107 | 14104947 | 13969205 |
| Total mapped | 8363998 (83.76%) | 9459906 (85.37%) | 12306499 (87.25%) | 12199271 (87.33%) |
| Multiple mapped | 350949 (3.51%) | 395255 (3.57%) | 555862 (3.94%) | 508780 (3.64%) |
| Uniquely mapped | 8013049 (80.25%) | 9064651 (81.8%) | 11750637 (83.31%) | 11690491 (83.69%) |
| Reads map to '+' | 4013086 (40.19%) | 4528897 (40.87%) | 5859016 (41.54%) | 5837557 (41.79%) |
| Reads map to '-' | 3999963 (40.06%) | 4535754 (40.93%) | 5891621 (41.77%) | 5852934 (41.9%) |
| Non-splice reads | 6790503 (68%) | 7603505 (68.62%) | 9917362 (70.31%) | 9784605 (70.04%) |
| Splice reads | 1222546 (12.24%) | 1461146 (13.19%) | 1833275 (13%) | 1905886 (13.64%) |

(1) Total reads：Count number of filtered reads (Clean data).

(2) Total mapped：Total number of reads could be mapped to the genome.

(3) Multiple mapped：Count number of reads that could be mapped to multiple sites in the reference genome.

(4) Uniquely mapped：Count number of reads that can be uniquely mapped to the reference genome.

(5) Reads map to '+', Reads map to '-': Count number of reads that mapped to positive strand or minus strand, respectively.

(6) Splice reads：Count number of reads that could be segmented and mapped to 2 exons, also named junction reads.
