# Supplementary material for: Fine mapping and RNA-Seq unravels candidate genes for a major QTL controlling multiple fiber quality traits at the T1 region in upland cotton
Source: BMC Genomics. 2016 Apr 19;17:295. doi: 10.1186/s12864-016-2605-6 (PMC4837631; doi:10.1186/s12864-016-2605-6)
Supplement: Additional file 9: Figure S2. — KEGG analysis results of differentially expressed genes at the 0 DPA ovule and 5 DPA fiber comparing RIL118 and Yumian1. (DOCX 195 kb) [file 12864_2016_2605_MOESM9_ESM.docx]

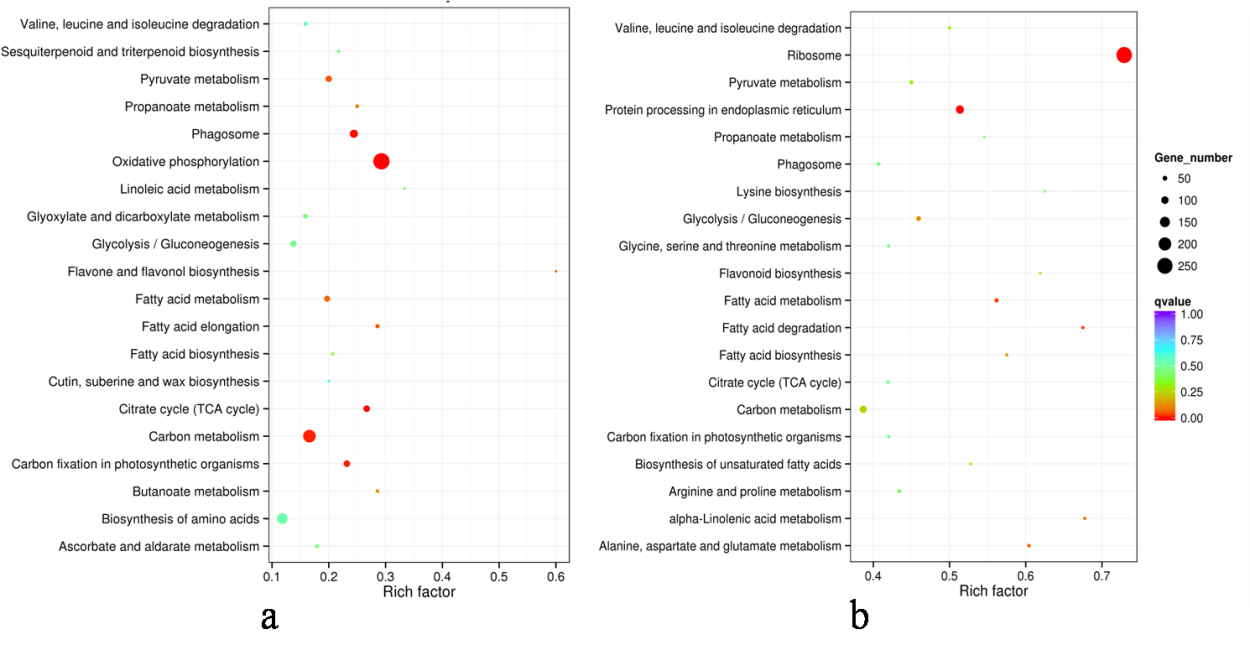


Figure 2. KEGG analysis results of differentially expressed genes at the 0 DPA ovule and 5 DPA fiber comparing RIL118 and Yumian1. Rich factor is the ratio between counts of differentially expressed genes and all annotated genes enriched in a certain pathway; qvalue is P value after multiple hypothesis testing correction with a range between 0 and 1. Twenty most significant pathways were plotted, when more than 20 pathways were identified. a, Results for the 0 DPA ovule. b, Results for the 5 DPA fiber.
